# Assessing the feasibility of free DNA for disaster victim identification and forensic applications

**DOI:** 10.1038/s41598-024-53040-0

**Published:** 2024-03-05

**Authors:** Wikanda Worrapitirungsi, Tikumphorn Sathirapatya, Poonyapat Sukawutthiya, Kornkiat Vongpaisarnsin, Pagparpat Varrathyarom

**Affiliations:** 1 Forensic Genetics Research Unit, Ratchadapiseksompotch Fund, Faculty of Medicine, Chulalongkorn University, Bangkok, Thailand; 2 Department of Forensic Medicine, Faculty of Medicine, Chulalongkorn University, Bangkok, Thailand; 3 Forensic Serology and DNA, King Chulalongkorn Memorial Hospital and Thai Red Cross Society, Bangkok, Thailand

**Keywords:** PCR-based techniques, Biological techniques

## Abstract

In tropical disaster victim identification (DVI) scenarios, challenging environmental conditions lead to accelerated DNA degradation in remains. To further enhance the utilization of leached DNA from tissue in the preservative solution (termed “free DNA”) as an alternative source, we incorporated new results by assessing its integrity in postmortem and decomposing cadavers preserved in DNA/RNA Shield™ and modified TENT, with silica-based purification (QIAquick^®^) for faster processing. The psoas muscle tissues of one decomposed and ten cadavers were preserved in each solution at 25 °C and 35 °C for 3 months. Free DNA efficiency was compared with individual reference samples for reliable results in quantity, quality, and STR profiles. The findings revealed that DNA/RNA Shield™ effectively preserves free DNA integrity for extended storage, while modified TENT is more suitable for short-term storage due to higher degradation levels. Moreover, the use of free DNA samples with massive parallel sequencing displays potential for forensic DNA analysis. Successful amplification of the mtDNA control region enables variant calling and heteroplasmy analysis while also serving as quality control using ACTB and enabling differentiation within the 16S rRNA region for microbiome analysis. The simplicity of handling free DNA for PCR-based forensic analysis adds to its potential for various applications, including DVI and field-based analysis of biological evidence.

## Introduction

The forensic identification of a human body poses significant challenges, particularly in mass disaster incidents. In such circumstances, the immediate collection of samples is required due to the rapid degradation of the body^1,2^. DNA degradation in biological samples occurs rapidly during decay, particularly when exposed to high temperatures and humidity. This degradation remains a challenge in terms of DNA amplification and results in a decrease in the success rate of STR profiling^3,4^. Therefore, careful collection and preservation of biological material are essential to ensure successful DNA analysis in forensic cases^5,6^. Storing samples at low temperatures or freezing is a common practice. However, in situations where facilities and electricity are limited, the development of various preservative solutions has addressed this challenge. These solutions offer the potential to store biological samples at different temperatures, thereby enabling DNA analysis across different sample types^7–11^.

Allen-Hall and McNevin^12^ examined various preservation techniques for disaster victim identification (DVI). They examined the effectiveness of various preservatives, including DESS solution, TENT buffer, and DNAgard^®^, in tissue preservation and leaching DNA, resulting in the presence of “free DNA”. However, it was noted that the quantity and quality of this free DNA might not be sufficient for successful genotyping or long-term stability. Another study by Sorensen^13,14^ revealed that the modified TENT buffer was the most efficient solution for preserving both fresh and decomposed tissues for up to 3 months, even in conditions of heat and humidity. To improve the condition, Holmes^15^ explored new methods to decrease the salt concentration in the modified TENT buffer and achieve faster DNA purification prior to STR typing, potentially enabling DNA profiles to be obtained without extensive extraction. However, further research is needed to evaluate the use of free DNA from alternative preservatives on postmortem or decomposed human tissues.

To date, free DNA evaluation has focused primarily on assessing the quantity of free DNA extracted from tissue samples preserved in different solutions and its potential to generate complete downstream STR profiles, mostly applied on fragment analysis via capillary electrophoresis (CE)^12–15^. There is still limited knowledge about the application of next-generation sequencing (NGS). Furthermore, free DNA represents a potential source from various perspectives. For example, the utilization of human β-actin (ACTB) as an endogenous reference gene in forensic research ensures standardized data for accurate gene expression comparisons across different samples^16,17^. Additionally, the use of 16S rRNA and internal transcribed spacer (ITS) markers in microbiome analysis improves species identification, contributing to our understanding of microbial communities in forensic research and their application in forensic investigations^18–20^. Therefore, it is crucial to provide additional supporting information on the utilization of free DNA from postmortem tissues within these research scopes.

Here, we assess the quantity and quality of free DNA released from postmortem and decomposed human muscle tissues into a preservative solution during a storage period of 3 months at 25 °C and 35 °C using silica-based purification (QIAquick^®^)^15^. The 35 °C incubation temperature used in this study simulates the conditions expected at mass disaster sites in tropical climates^12^. Additionally, the quality of free DNA is evaluated using various techniques, including STR profiling using both fragment analysis via CE and NGS, analysis of two hypervariable regions (HV1 and HV2) in the mitochondrial DNA control region using NGS, and amplification of ACTB, 16S rRNA, and ITS. The objective of this study is to explore the feasibility of applying free DNA derived from postmortem and decomposed tissues as a novel and effective resource in DVI processes and forensic applications.

## Methods

### Ethical approval

All procedures were performed in accordance with the guidelines of the Helsinki Declaration. This study did not involve the collection of identifiable private information and is exempt from the need for ethical approval under 45CFR 46.101(b) by the Institutional Review Board (IRB) of the Faculty of Medicine, Chulalongkorn University (IRB.No.327/64).

### Sample collection and preservation

**Figure 1:**
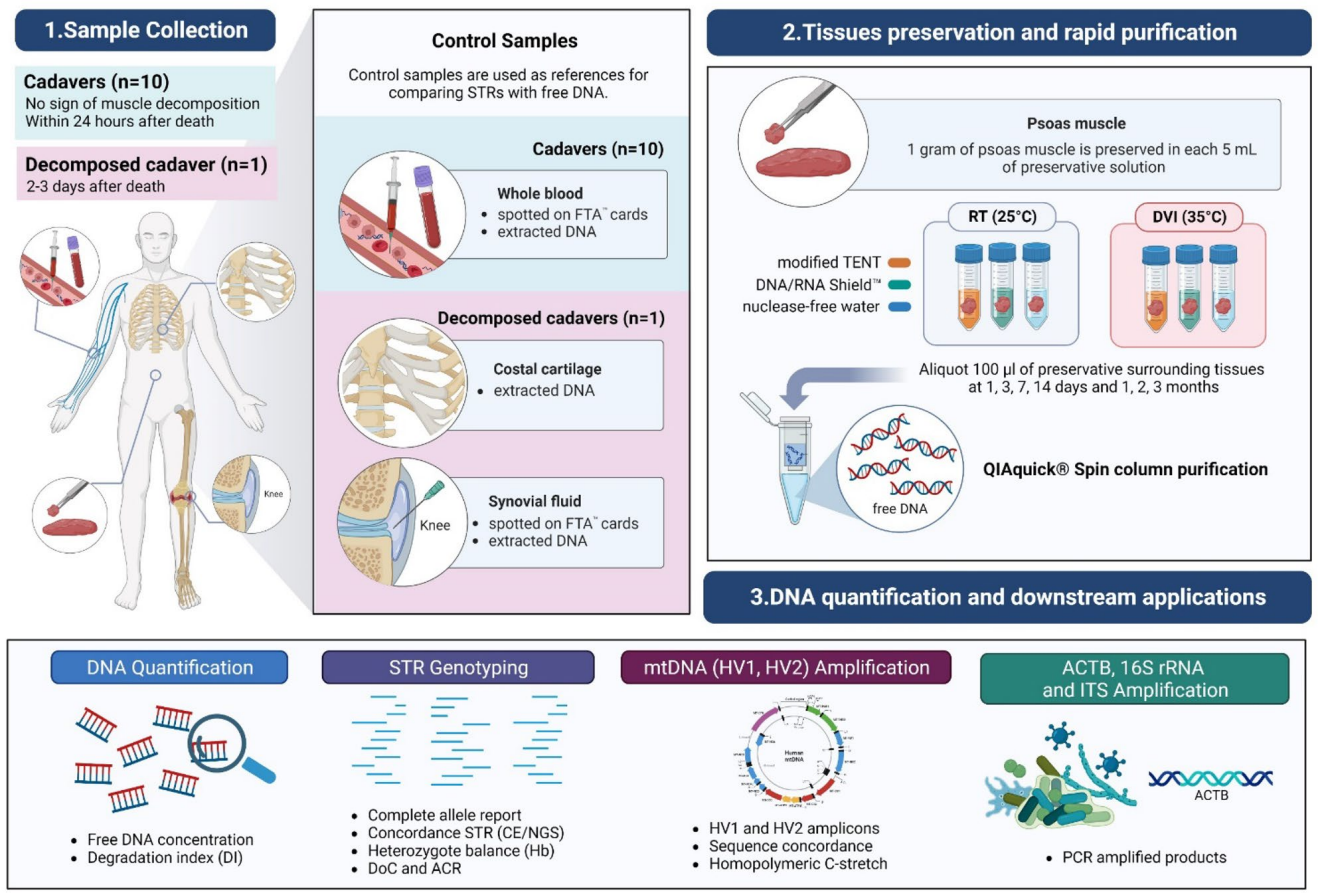
Sampling and experimental setup: Psoas muscle was collected from a total of ten cadavers and one decomposed cadaver. Blood samples from all cadavers were spotted onto FTA^®^ cards, serving as reference samples, as well as costal cartilage and synovial fluid for decomposed cadavers. The collected muscle tissues were subjected to three different storage treatments involving various solutions, storage times, and temperatures. Specifically, these samples were treated with modified TENT buffer, DNA/RNA Shield™, and nuclease-free water at 25 °C and 35 °C, respectively. At intervals of 1, 3, 7, and 14 days and 1, 2, and 3 months, aliquots were taken from the preservative surrounding the samples. These aliquots were then purified for subsequent quantification, STR genotyping, HV1/HV2 mtDNA analysis, and amplification of ACTB, 16S rRNA, and ITS.

The samples included in this study comprised muscle tissue, blood, costal cartilage, and synovial fluid. Psoas muscle was collected from ten human cadavers that met specific inclusion criteria. First, the cadavers were required to have a postmortem interval (PMI) within 24 h. Second, there should be no signs of muscle decomposition on examination by gross morphology. Additionally, to represent a decomposing cadaver sample, a body with a PMI of 2–3 days was included. All the samples used in this research were provided by the Department of Forensic Medicine, Faculty of Medicine, Chulalongkorn University, Bangkok, Thailand. The muscle samples were carefully dissected into sections weighing ~1 g. The sections were then immersed in 5 mL of three different preservative solutions. These solutions included modified TENT buffer [(10 mM Tris (Merck), 10 mM EDTA (Merck), 1 M NaCl (Sigma-Aldrich), 2% Tween 20 (Sigma-Aldrich); 100 mL, pH 8.0)], DNA/RNA Shield™ (Zymo Research) and nuclease-free water (Apsalagen) used as a control. Samples were stored at 25 °C and 35 °C for specific time intervals: 1, 3, 7, 14 days and 1, 2, and 3 months (Fig. 1). Moreover, reference samples were incorporated, including whole blood spotted on Whatman^®^ FTA^®^ cards from ten cadavers, as well as costal cartilage and synovial fluid from the decomposed body.

### Genomic extraction and rapid purification

To ensure accuracy and precision, aliquots of 100 µL were duplicated from each preservative surrounding the tissues and subsequently transferred to individual 1.5 mL sterile microcentrifuge tubes. Free DNA purification from each tube was conducted using the QIAquick^®^ PCR Purification Kit (QIAGEN), following the manufacturer’s protocol. Then, by pooling the duplicate purified free DNA in a single tube, each purified sample was stored at − 20 °C. For reference specimens, the cleaned dried costal cartilage was finely powdered utilizing the Precellys^®^ Evolution Homogenizer (Bertin Technologies). To extract DNA from the powdered costal cartilage, the PrepFiler™ BTA Forensic DNA Extraction Kit (Applied Biosystems) was employed, following the manufacturer’s instructions.

Regarding the synovial fluid, a portion was directly spotted on Whatman^®^ FTA^®^ cards for a direct PCR reaction. Regarding the remaining synovial fluid, extraction was performed using the QIAamp^®^ Blood Mini Kit (QIAGEN). The extracted DNA from both the costal cartilage and the synovial fluid was stored at − 20 °C until further downstream analysis.

### DNA quantification

All purified free DNA samples were quantified using the Quantifiler™ HP DNA Quantification Kit (Applied Biosystems) on a QuantStudio™ 5 Real-Time PCR System (Applied Biosystems). The data were then analyzed using the HID Real-Time PCR Analysis Software v1.2. The correlation coefficient R^2^ ≥ 0.99 was used. The internal PCR control (IPC) value was monitored for the presence of PCR inhibitors. Additionally, the degradation index (DI) was calculated to evaluate the degree of degradation by determining the ratio of small to large DNA concentration. Furthermore, the extracted DNA samples underwent an assay using the QuantiFluor^®^ ONE dsDNA System (Promega) on the Quantus™ Fluorometer (Promega), following the manufacturer’s protocol.

### STR genotyping with capillary electrophoresis

The samples were genotyped using the PowerPlex^®^ Fusion 6C System (Promega). DNA amplification was carried out using the GeneAmp^®^ PCR System 9700 (Applied Biosystems) or the ProFlex™ 3×32-Well PCR System (Applied Biosystems). The PCR products were detected using an ABI 3500 or 3500xL Genetic Analyzer (Thermo Fisher Scientific) equipped with a 36 cm capillary array and POP4^®^ polymer from Applied Biosystems. Initial fragment sizing and allele calling were performed using GeneMapper^®^ ID-X software version 1.4 (Applied Biosystems) with a 50 RFU analytical threshold. STR loci with heterozygote balance (Hb) < 0.7 were considered imbalanced.

### STR genotyping using MiSeq FGx™ sequencing

Libraries were prepared using the ForenSeq™ DNA Signature Prep Kit Primer Mix A (Verogen), using a total DNA input of 1 ng, and performed according to the manufacturer’s protocol. The sequencing library was then quantified using NEBNext^®^ Library Quant Kit for Illumina^®^ (NEB) before being sequenced on the MiSeq FGx™ Forensic Genomics System (Illumina) according to the manufacturer’s instructions. The sequencing run quality metrics were all within the recommended range.

### mtDNA amplification and sequencing

Two hypervariable regions (HV1 and HV2) of human mtDNA were separately amplified using primers (HV1: L15977/H16401; HV2: L29/H408). The samples were amplified using a ProFlex™ 3×32-Well PCR System (Applied Biosystems). HV1 and HV2 PCR products were quantified using the QIAxpert^®^ System (QIAGEN), and the sample concentrations were subsequently normalized. The mtDNA library preparation was generated using the Illumina^®^ DNA Prep method (Illumina). All libraries were subjected to the MiSeq FGx™ Reagent Micro Kit (Illumina) and sequenced on the MiSeq FGx™ Sequencing System (Illumina). The sequencing data was visualized using the Universal Analysis Software 2.0 module (Verogen). The sequencing results were aligned to the revised Cambridge reference sequence (rCRS) for variant calling. The concordance of variants between each sample was observed.

### ACTB, 16S rRNA and ITS amplification

All primers used in this study for human ACTB, 16S rRNA, and ITS are presented in Supplementary Table S1. Free DNA from fresh tissue samples from one cadaver, including decomposed tissues under all preservative conditions, was selected for the amplification of ACTB. Only free DNA from decomposed tissues preserved in DNA/RNA Shield™ amplified 16S rRNA and ITS primers. PCR reactions were performed for each sample in 50 μL volumes, consisting of 25 μL of KOD One™ PCR Master Mix, 1.5 μL of 10 μM primer, 2 ng of DNA from each sample and molecular-grade water as a negative control. Subsequently, all PCR products were separated using the QIAxcel^®^ DNA High-Resolution Kit (QIAGEN) on the QIAxcel^®^ Advanced System (QIAGEN) instrument.

### Statistical analysis

The effects of three variables (temperature, preservative and time) on free DNA concentration at various time points were analyzed using a General Linear Model-Repeated Measures in IBM^®^ SPSS^®^ software version 29. A significance level of *p* < 0.05 was considered statistically significant.

## Results

### Free DNA concentration in each preservative

**Figure 2:**
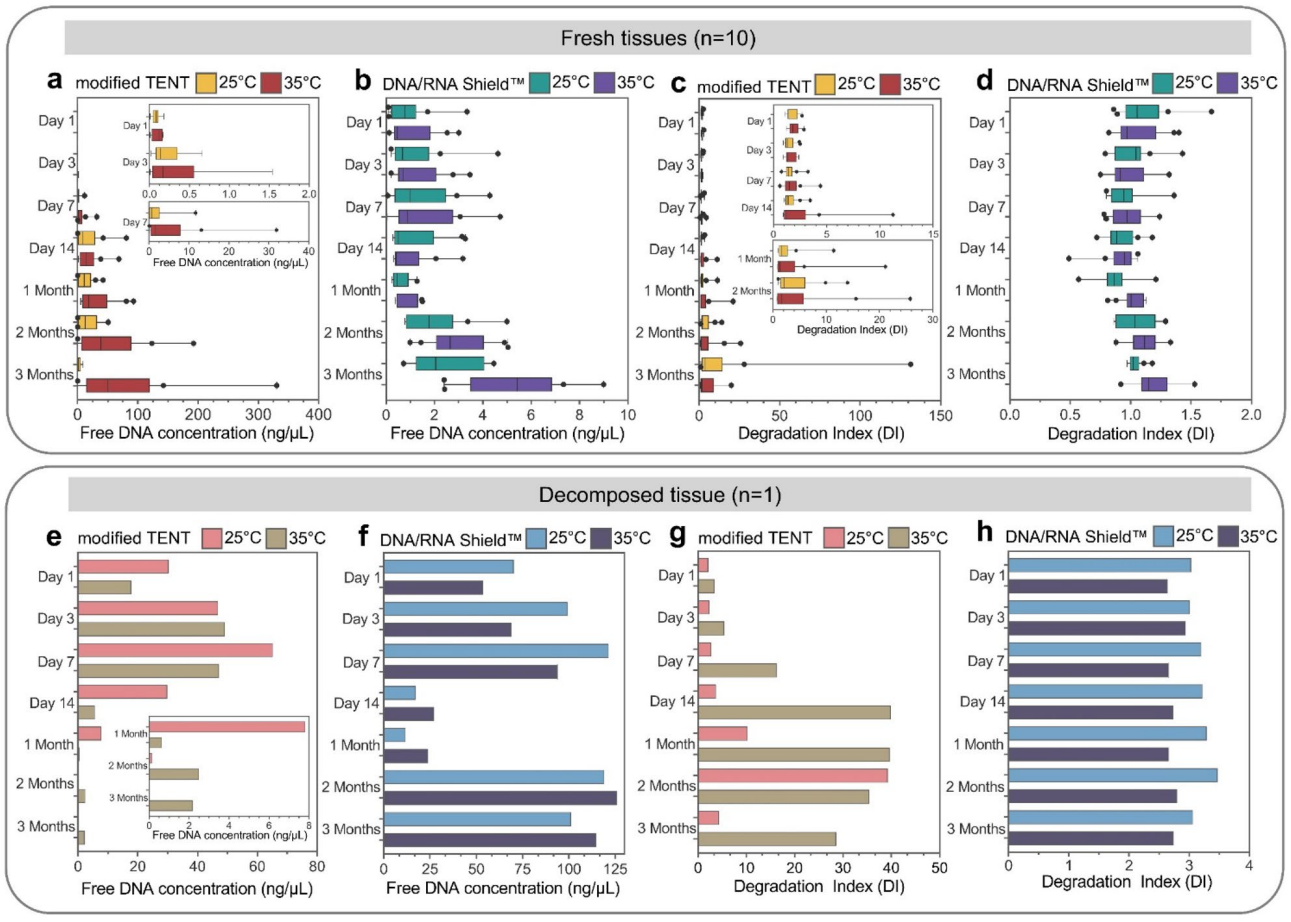
Box-and-whisker plots depicting total free DNA concentration (ng/µL) and degradation index of fresh tissues (n = 10) preserved in modified TENT (**a**,**c**) and DNA/RNA Shield™ (**b**,**d**) at 25 °C and 35 °C for up to 3 months. Outliers are represented by dots. Bar graphs illustrating total free DNA concentration (ng/µL) and degradation index of decomposed tissue (n = 1) stored in modified TENT (**e**,**g**) and DNA/RNA Shield™ (**f**,**h**) at 25 °C and 35 °C for up to 3 months.

The results of free DNA quantity from fresh tissues were summarized in Fig. 2a,b, indicating increasing trends over time for both modified TENT and DNA/RNA Shield™ preservatives, except for modified TENT at 25 °C, which decreased at 3 months. The average free DNA quantities ranged from 0.1 to 19 ng at 25 °C and 0.1 to 85 ng at 35 °C for modified TENT, and 0.6 to 2.4 ng at 25 °C and 0.8 to 5.3 ng at 35 °C for DNA/RNA Shield™, respectively. Statistical analysis revealed a significant increase in free DNA quantity from modified TENT at both 25 °C and 35 °C on day 14 (*p* < 0.05, *p* < 0.01, respectively) (Supplementary Figure S1). In contrast, free DNA yields from DNA/RNA Shield™ remained relatively stable from day 1 to 1 month at both 25 °C and 35 °C. Significant increases were observed after 2 months at both 25 °C (*p* < 0.01) and 35 °C (*p* < 0.001).

Notably, the statistical analysis demonstrates that temperatures, preservatives, and times strongly influence the quantity of free DNA from fresh tissues under various conditions (Supplement Table S2). Furthermore, the free DNA quantity from decomposed tissue, as shown in Fig. 2e–f, exhibited an initial steady increase from day 1 to day 7, followed by a dramatic decrease on day 14 for both modified TENT and DNA/RNA Shield™ preservatives. For modified TENT (Fig. 2e), it continued to decrease rapidly after day 14, while for DNA/RNA Shield™ (Fig. 2f), the quantity remained constant until 1 month and then gradually increased from 2 to 3 months.

Moreover, we observed DI values to assess the preservation of sample integrity in terms of both free DNA quantity and quality under different storage conditions. None of the free DNA samples indicated PCR inhibition. The average DI values for free DNA from fresh tissues preserved in modified TENT ranged from 2 to 5, except for 3 months at 35 °C, where it increased to 19 (Fig. 2c). For decomposed tissues at 25 °C, the DI value initially increased at 1 month, reached its peak at 2 months, and then declined at 3 months. In comparison, at 35 °C, the DI value increased on day 7, peaked on day 14, and continued to decline until 1 month (Fig. 2g). The average DI values from all samples preserved in DNA/RNA Shield™ remained relatively stable over time for both fresh tissues (ranging from 0.9 to 1.1 at 25 °C and 0.9 to 1.2 at 35 °C) and decomposed tissues (ranging from 3.1 to 3.5 at 25 °C and 2.7 to 2.8 at 35 °C) at 25 °C and 35 °C (Fig. 2d,h).

### STR genotyping

**Figure 3:**
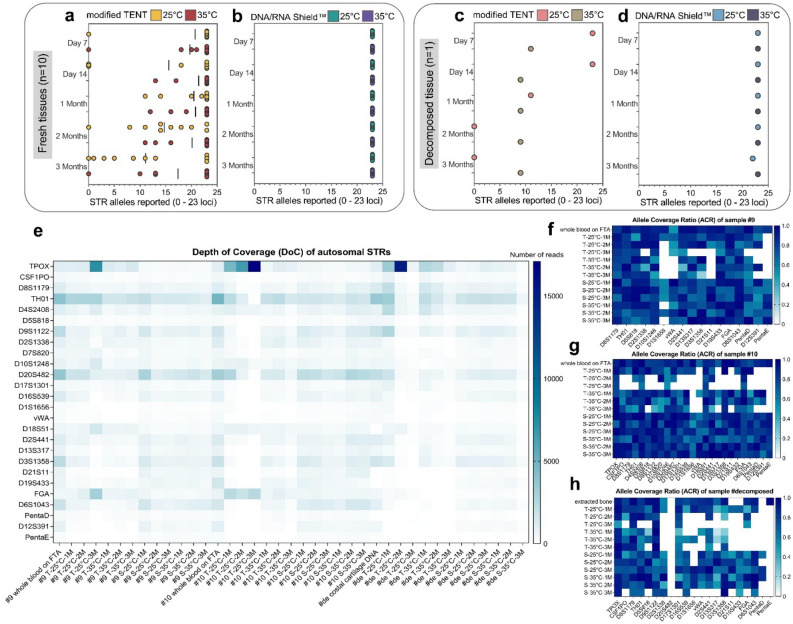
The scatterplot depicts the success of STR allele reporting (only autosomal 0–23 STR loci) for (**a**) free DNA in modified TENT from fresh tissues (n = 10), each bar in the graph represents the mean of the results at each time (**b**) free DNA in DNA/RNA Shield™ from fresh tissues (n = 10) (**c**) free DNA in modified TENT from decomposed tissue (n = 1) and (**d**) free DNA in DNA/RNA Shield™ from decomposed tissue (n = 1). The STR results of free DNA purified from all preservatives stored for up to 3 months were compared to the allele calling from whole blood, synovial fluid, or costal cartilage as reference STR profiles. Heat maps with continuous color shading (**e**) show the depth of coverage (DoC) of autosomal STRs from the free DNA samples under various conditions detected on the NGS platform from Illumina, MiSeq FGx™ Forensic Genomics System. The loci are listed in ascending order of autosomal STR amplicon size, from smallest to largest. Sections (**f**–**h**) show the allele coverage ratio (ACR) of the heterozygous alleles from each sample. Colors represent shades of blue based on allelic imbalances, while white indicates the dropout of the heterozygous allele. The loci are arranged from left to right in ascending order of autosomal STR amplicon size.

The numbers of reportable alleles observed in PowerPlex^®^ Fusion 6C are shown in Fig. 3a–d. We calculated the percentage of correctly called alleles by comparing 23 autosomal STR loci with their reference samples, as mentioned in the methods. Overall, the STR profiles obtained from free DNA preserved in modified TENT and DNA/RNA Shield™ exhibited concordance with the genotypes obtained from their respective reference samples. However, the quality of the resulting STR profiles depended on the quality of the free DNA, as indicated by the DI value. Although none of the purified free DNA samples exhibited PCR inhibition, there were variations in DI between the preservatives and, over time, up to 3 months. A high DI value suggests that larger DNA fragments may be more degraded and less reliable in producing accurate results in STR analysis compared to smaller DNA fragments. We observed that free DNA purified from fresh tissues preserved in DNA/RNA Shield™ consistently exhibited complete profiles from day 7 to 3 months (Fig. 3b), regardless of the storage conditions at either 25 °C or 35 °C. However, free DNA preserved in modified TENT predominantly produced partial profiles with a higher number of detected STR loci at 35 °C (80–93%) compared to room temperature (45–80%). The analysis of free DNA purified from decomposed tissues and preserved in DNA/RNA Shield™ indicated complete profiles, except for 3 months at 25 °C, where 96% of alleles were reported (Fig. 3d). Conversely, free DNA preserved in modified TENT exhibited partial STR profiles (39–48%) at 35 °C, while full STR profiles were obtained at room temperature, except for 1 month, where only 48% of alleles were reported, and no STR profiles were observed on 2 months and 3 months.

The heterozygote balance (Hb) for autosomal STR loci of fresh tissues (n = 10) and decomposed tissue (n = 1) under various conditions is presented in Supplementary Figures S2–S5. Our findings indicate that the majority of Hb values across profiles recovered from free DNA purified from DNA/RNA shield remained consistently balanced (Hb > 0.7), whereas modified TENT showed more imbalanced loci (Hb < 0.7).

Two free DNA samples purified from fresh tissues (samples #9 and #10) and one free DNA sample from decomposed tissue were sequenced using the MiSeq FGx™ System. We selected two cadaver samples, sample #9 and sample #10, as representatives of the fresh tissue samples group. This decision was based on their characteristics falling within the median range in terms of quantity and quality for sample #9, while sample #10 exhibited the highest DI and was utilized to evaluate the performance of the NGS method under challenging conditions. We subjected all sequencing runs to analysis using the ForenSeq™ UAS, followed by a comparison of allele reports across 22 shared autosomal STR loci with those obtained through the PowerPlex^®^ Fusion 6C assay. Table 1 displays the detection rates for shared autosomal STR loci using both methods. Six markers from ForenSeq™ sequencing have been excluded from the analysis to ensure precision in the calculations.

**Table 1:** Comparison of the qualification and detection rates of free DNA samples for the shared autosomal STR loci between the capillary electrophoresis (CE) and next-generation sequencing (NGS) methods.

Samples	Preservatives	Temperatures (°C)	Storage times (month)	Degradation Index	STR detection rate (CE methods) (%)	STR detection rate (NGS methods) (%)
sample #9 fresh tissues	modified TENT	25	1	1.5	100	91
			2	1.4	86	86
			3	6.0	32	55
		35	1	3.6	82	68
			2	2.5	68	77
			3	4.0	55	73
	DNA/RNA Shield™	25	1	1.2	100	95
			2	1.2	100	95
			3	1.2	100	95
		35	1	1.1	100	91
			2	1.1	100	95
			3	1.5	100	91
sample #10 fresh tissues	modified TENT	25	1	4.4	59	68
			2	9.9	36	36
			3	131.4	14	18
		35	1	1.5	100	86
			2	2.3	95	82
			3	8.7	50	59
	DNA/RNA Shield™	25	1	0.8	100	95
			2	0.9	100	95
			3	1.0	100	95
		35	1	1.0	100	95
			2	0.9	100	95
			3	1.3	100	95
sample #decomposed tissues	modified TENT	25	1	10.3	55	82
			2	39.3	5	50
			3	4.3	0	27
		35	1	39.7	45	59
			2	35.5	45	59
			3	28.6	45	41
	DNA/RNA Shield™	25	1	3.3	100	86
			2	3.5	100	82
			3	3.1	95	91
		35	1	2.7	100	91
			2	2.8	100	86
			3	2.7	100	50

Six markers (including SE33, D4S2408, D9S1122, D20S482, D17S1301 and D6S1043) from ForenSeq™ sequencing have been excluded from the analysis to ensure precision in the calculations.

In summary, the ForenSeq™ sequencing of reference samples and free DNA sequencing from both fresh and decomposed tissues demonstrated overall concordance with genotypes obtained from PowerPlex^®^ Fusion 6C assays (Supplementary Tables S3–S6). As the DI increased, the detection rates of STRs decreased in both platforms. Our findings suggest that the efficiency of preservatives, time and temperature affects the success rates of samples. DNA/RNA Shield™ exhibited a higher STR detection rate than modified TENT when using the capillary electrophoresis (CE) method, with a success rate of 100% for fresh tissues and 95–100% for decomposed tissues. However, with the NGS method, dropout allele phenomena were observed in DNA/RNA Shield™, and the 3-month decomposed tissues showed more absent alleles compared to the CE method (Table 1).

We found that the results for D22S1045 from the NGS method showed an inconclusive result (INC) or a complete absence of the allele in all runs. However, the NGS method demonstrated superior performance, particularly in the case of modified TENT samples, especially for free DNA extracted from decomposed tissues (Supplementary Table S3). The NGS method exhibited higher sensitivity in detecting smaller amplicons, such as the CSF1PO and D8S1179 loci, compared to the CE method, which showed inconsistent results. In addition, we also evaluated the performance of the autosomal STRs using the allele sequencing reads, the values of the average depth of coverage (DoC) and allele coverage ratio (ACR). The DoC for autosomal STRs obtained from two free DNA samples extracted from fresh tissues (samples #9 and #10) and one free DNA sample from decomposed tissues on the NGS platform is presented in Fig. 3e. Figure 3f–h exhibit the interlocus allele coverage ratio of STRs calculated individually as the lowest coverage of homozygous loci divided by the highest coverage of each sample. The DoC per marker ranged from a minimum of 67.31 reads for PentaE to a maximum of 2123.53 reads for TPOX.

Furthermore, our study found that Penta E had the lowest ACR at 0.27, while TPOX had the highest ACR at 0.86. We suggest that ACR tends to decrease over time in all samples. Most of the free DNA from the fresh tissue-derived DNA/RNA Shield™ samples exhibited a high interlocus balance and an ACR greater than the general STR intralocus balance threshold (0.6) set by the ForenSeq™ UAS software.

### mtDNA sequencing

We selected sample #10 and a decomposed sample for mtDNA (HV1 and HV2) sequencing by NGS. Sample #10 was chosen because it exhibited the highest DI among fresh tissue cadaver samples. We opted for mtDNA sequencing as an alternative forensic marker for degraded samples, utilizing its multicopy nature for human identification^21^. Sequencing results were evaluated by comparing the mtDNA profiles of free DNA with their respective extracted DNAs (Supplementary Table S7). All sequence variants found in both sources were in concordance. The common base pair range of sequenced samples is 15998–16400 for HV1 and 29–408 for HV2. The most common polymorphic positions are 73 A–G, 263 A–G, 16223 C–T point mutation and 315.1 C insertion, which are presented in every individual.

**Figure 4:**
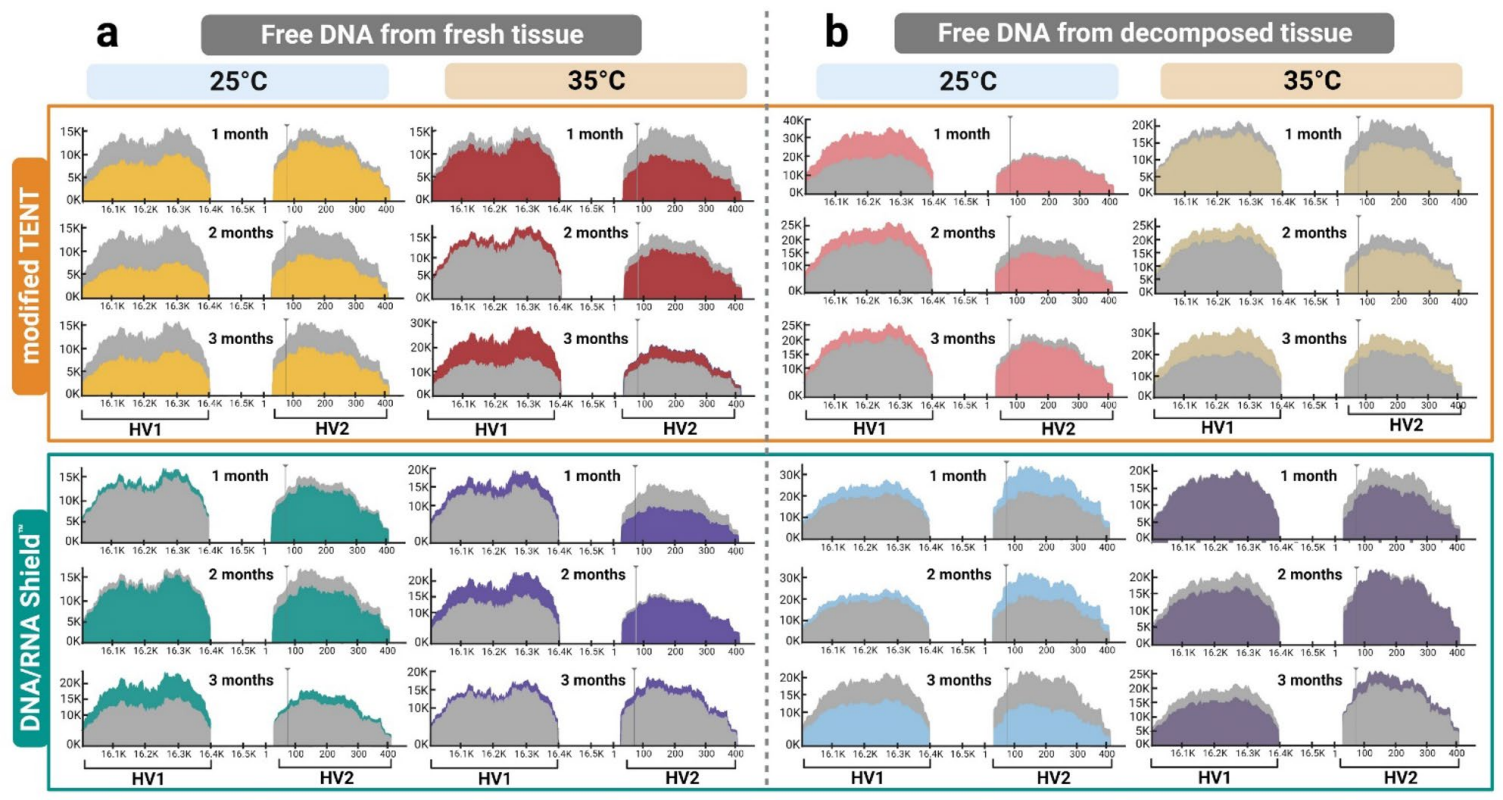
Comparing the sequencing coverage of the HV1 and HV2 regions on the NGS platform between free DNA purified from (**a**) fresh tissues (sample #10) and (**b**) decomposed tissues under various conditions. Positions of HV1 and HV2 are plotted along the x-axis, and the y-axis shows coverage. The gray histogram represents a control for free DNA obtained from both fresh and decomposed tissues. This control is appropriately derived from the extracted DNA from blood and the extracted DNA from costal cartilage. The color-overlaid gray histograms illustrate the coverage of the selected sample conditions.

Furthermore, we investigated the homopolymeric C-stretch within the HV1 and HV2 regions across all samples subjected to various conditions. Our findings revealed the absence of heteroplasmy in all samples. Detailed information on the C-stretch sequence patterns at the nucleotide positions 16184–16193 and 303–315 is defined in Supplementary Table S8. In our study, we observed concordance between the HV1 and HV2 sequences obtained from the same samples under different experimental conditions. Moreover, we also identified variations in the sequence coverage for each sample condition, ranging from 15,000 to 30,000 reads (Fig. 4).

We found that the coverage patterns varied depending on the temperature, tissue condition and the type of preservative solutions. The coverage of the HV1 and HV2 regions in free DNA from fresh tissues at 25 °C using modified TENT was lower than that of the control, with a decreasing trend over time. However, when DNA/RNA Shield™ was used, the coverage was slightly higher than the control, except for HV2 at 2 months. For free DNA from fresh tissues at 35 °C with modified TENT, the coverage of HV1 initially exceeded the control, decreased slightly at 2 months, and significantly increased to 30K at 3 months, while the coverage of HV2 was lower than the control at 1 and 2 months but higher at 3 months. When DNA/RNA Shield™ was used, the HV1 regions consistently exhibited higher coverage compared to the control, while the HV2 regions remained relatively similar to the control, except at 2 months when it was lower (Fig. 4a). In decomposed tissues at 25 °C using modified TENT, HV1 coverage was consistently higher than the control, while HV2 coverage remained similar, except at 2 months. With DNA/RNA Shield™, both HV1 and HV2 had higher coverage than the control, maximizing at 30K for HV2 at 1 to 2 months but decreasing at 3 months (Fig. 4b).

### ACTB, 16S rRNA and ITS amplification

Amplified ACTB products were detected across various sizes in free DNA samples from fresh tissues preserved in DNA/RNA Shield™ at different time points (Day 7, Day 14, and 1–3 months) and temperatures (25 °C and 35 °C). However, only ACTB’s amplified free DNA was detected with modified TENT on Day 7, 14, and 1 month at 25 °C using ACTB1 and ACTB2 primers. Among the free DNA from decomposed samples, only amplified ACTB from the free DNA preserved in DNA/RNA Shield™ was found at various time points and temperatures, while the free DNA preserved using modified TENT was not detectable. Furthermore, we performed analyses on free DNA from decomposed tissues preserved in DNA/RNA Shield™, specifically focusing on the 16S rRNA and ITS regions. Our investigation revealed successful amplification of PCR products for the 16S rRNA region at all tested time points, both at 25 °C and 35 °C. However, no PCR products were detected for the ITS region (Supplementary Table S9).

## Discussion

### Environmental factors affect the quantity and quality of free DNA

The statistical analysis of free DNA quantity from fresh tissues under various conditions reveals that each variable (temperatures, preservatives, times) significantly impacts the quantity of free DNA (Supplement Table S2). However, when examining the interaction between times and temperatures on the amount of free DNA through pairwise comparisons, no significant differences were observed except at 3 months (*p* < 0.05). This indicates that the effect of time and temperature on free DNA quantity is independent of each other. Our study demonstrates the effectiveness of both modified TENT and DNA/RNA Shield™ in maintaining free DNA integrity. Nonetheless, DNA/RNA Shield™ stands out as the preferred option for long-term storage, given its superior ability to maintain free DNA integrity in both tissue types.

Our findings support a previous study indicating that modified TENT effectively preserves free DNA from fresh tissues collected in the field at room temperature^15^. Conversely, our results show contrasting outcomes when storing free DNA from decomposed tissue in modified TENT at 35 °C, contradicting the previous study^12,14^. This disparity can be attributed to the unique conditions of our study. All the cadavers we examined were collected from a scene situated in Thailand, where the mean temperature was 30 °C, contributing to a higher rate of tissue decomposition compared to the previous study^22^. The variations in free DNA quantity observed in this study can be attributed both to the ratio of buffer volume to tissue amount and the inherent heterogeneity of individual muscle cadavers. Additionally, variability in the subsampling of the preservation solution at each time point could introduce potential confounding factors into the results.

Moreover, we observed differences in the characteristics of muscle tissues after storage for up to 3 months. Tissues preserved in modified TENT showed a gradual disintegration over time, whereas those preserved in DNA/RNA Shield™ exhibited a color change but maintained their structural integrity immediately after preservation, which persisted for the entire 3-month period. The presence of specific chemicals, potentially including oxidizing agents, in DNA/RNA Shield™ is hypothesized to be associated with this phenomenon. When using QIAquick^®^ for purification, we observed that an aliquot of preservative from modified TENT after 2–3 months resulted in the appearance of tissue fractions on the silica membrane inside the spin column. This interruption could potentially affect the elution buffer during free DNA purification. On the contrary, when an aliquot of DNA/RNA Shield™ was used, it easily passed through the column, facilitating a smoother purification process.

The differential performance of modified TENT and DNA/RNA Shield in leaching DNA from fresh and decomposed samples is attributed to their distinct mechanisms of action. The modified TENT’s enhanced ability to disrupt cell membranes in fresh samples facilitates the release of more free DNA, while DNA/RNA Shield’s protective coating effectively safeguards free DNA from degradation in decomposed samples. This is evident in our findings, which indicate that modified TENT exhibits a limited ability to preserve free DNA beyond 2 months, leading to degradation in decomposed tissues. Conversely, DNA/RNA Shield™ effectively preserves free DNA for up to 3 months. These observations suggest that modified TENT is the optimal choice for fresh sample DNA extraction, while DNA/RNA Shield™ is better suited for degraded samples.

Previous studies have shown that the concentration of NaCl in the TENT buffer plays a crucial role in free DNA yield and protection against degradation during long-term storage^12–15^. NaCl is believed to establish an isotonic environment, preserve structural integrity, and stabilize tissue samples^23,24^. However, with longer storage durations, there is an increased probability of water evaporation from the preservative solution, resulting in a higher salt concentration and potential pH changes that can affect DNA integrity and stability. Additionally, endonucleases and exonucleases can rapidly break down DNA within cells^25–27^, plus oxidative damage from free radicals and hydrolysis, especially in acidic water^28,29^, compromises DNA integrity. Numerous previous studies have examined the impact of high NaCl concentrations on DNA structure and the potential increase in DNA degradation rates^30–32^. Interestingly, recent research findings highlight that DMSO and NaCl may not contribute significantly to the effectiveness of DESS preservative, and only EDTA directly preserves high molecular weight DNA^33,34^.

### The impact of free DNA integrity on the completeness and dropout of STR profiles

Our results clearly demonstrate that DNA/RNA Shield™ more effectively preserves the integrity of free DNA in the solution compared to the modified TENT buffer^35^. The quantity and quality of free DNA in DNA/RNA Shield™ remained relatively stable under various conditions, indicating its effectiveness in preventing DNA from tissues from leaching into the solution. Moreover, the quantity and quality of free DNA preserved in DNA/RNA Shield™ proved to be sufficient for generating comprehensive coverage of the highly discriminatory STR markers using both CE and NGS methodologies. In contrast, modified TENT appeared to promote the release of free DNA into the solution, as indicated by a gradual increase in free DNA quantity over time, unlike DNA/RNA Shield™. However, modified TENT was less effective in preserving the integrity of free DNA, resulting in inferior coverage of STR profiles compared to DNA/RNA Shield™. To preserve free DNA in both fresh and decomposed tissues for long-term storage in DVI operations, we recommend using DNA/RNA Shield™. The DNA/RNA Shield™ consistently outperformed modified TENT in generating more complete and balanced STR profiles using both CE and NGS methods. Whereas NGS offers a more advantageous technique for analyzing highly degraded free DNA from modified TENT samples that produced unsatisfactory STR results using the CE method (Supplementary Table S4–S5). The enhanced sensitivity of the NGS method enabled the generation of more STR profiles from free DNA from modified TENT samples stored at both 25 °C and 35 °C compared to CE. This additional information provided by NGS can be invaluable for STR profile interpretation.

Nevertheless, DNA/RNA Shield™ has a 12-month shelf life and may necessitate restocking, which could be costly for some laboratories. Therefore, the development of a simple in-house solution like modified TENT remains valuable for unidentified human remains in DVI. This is due to its cost-effectiveness, the ready availability of chemicals in most laboratory settings, and its ability to generate STR profiles suitable for short-term storage (1–2 months for fresh tissues and less than 1 month for decomposed tissue).

Moreover, we identified limitations in genotyping the D22S1045 loci using NGS with free DNA samples, as indicated by an increase in the inconclusive result (INC). It is important to note that the exclusion of D22S1045 from the MiSeq FGx™ System raises concerns about its performance^36,37^. The dropouts of D22S1045 at lower DI values may not be attributed solely to degradation but could also be attributed to poor marker performance^38^. Additionally, our findings revealed a correlation between the DI value and DoC, particularly in the case of small amplicon markers, such as TPOX, observed in decomposed tissues compared to fresh tissues. This suggests that DNA integrity influences the DoC, with a higher DI associated with a greater DoC for small amplicon markers. Conversely, large amplicon markers like PentaE exhibit lower DoC. This distinction becomes particularly evident in decomposed tissues, underscoring the importance of considering tissue conditions when selecting suitable markers for analysis.

Although modified TENT appears to leach more DNA from the sample compared to DNA/RNA Shield™, its ability to preserve DNA quality may be less effective. However, these seemingly contradictory results could offer distinct advantages for specific applications or priorities. For instance, if maximizing DNA yield for identity SNP analysis is the primary goal, the increased DNA leaching observed with modified TENT could be advantageous. Our NGS results demonstrated that the greater quantity of free DNA from modified TENT increased the likelihood of successfully identifying specific SNPs, making it suitable for applications where DNA yield is a critical factor (data not shown). Therefore, the choice between modified TENT and DNA/RNA Shield™ depends on the specific goals and requirements of the analysis.

### The potential utilization of free DNA as an alternative source for various forensic analyses

In the highly degraded sample, mtDNA analysis can be used to extract forensically significant information^39^. We successfully amplified the control region of both fresh tissue and decomposed tissue preserved for 3 months. Our results demonstrated concordance in variant calling when comparing DNA samples extracted from free DNA and DNA extracted from the same individual. However, the sequencing coverage of modified TENT and DNA/RNA Shield™ differed under various conditions. The sequence quality of the read coverage depth obtained from NGS platforms, which has been observed in some studies, plays a crucial role in determining the sensitivity of detecting and resolving heteroplasmic sites^40,41^. The optimization carried out in the studies aimed to evaluate the pattern of heteroplasmic mutations, including low-level heteroplasmy information (below 10%)^42^. They found that a read depth of >2000 is ideal for robust detection of variants at or below 2%. This level of read depth ensures accurate identification of subtle variations in mitochondrial DNA^43,44^. We reveal that free DNA can serve as a promising alternative source for investigating mtDNA heteroplasmy analysis. This expands the options available to forensic investigators when other sample types are limited.

In our study, we explored the free DNA to differentiate individuals within the 16S rRNA and ITS regions, which are important for studying the microbiome and metagenomic species^45,46^. Our results showed successful amplification of PCR products from the 16S rRNA region at various time points and temperatures. However, no PCR products were detected for the ITS region. This successful amplification of PCR products from free DNA, specifically in the ACTB and 16S rRNA regions, highlights the potential of using free DNA in forensic cases for individual identification and microbiome analysis^47–50^. Importantly, our study did not observe cross-contamination issues with the free DNA samples. However, it remains crucial to prevent contamination during sample collection and handling in the field. Wearing gloves, using sterilized plastic consumables and employing disposable instruments or thorough cleaning of reusable instruments are essential precautions.

## Conclusion

Our research demonstrates the successful utilization of free DNA as an alternative source for DVI and various forensic analyses. The consistent and concordant results from free DNA validate its reliability against individually extracted DNA, the forensic gold standard. DNA/RNA Shield™ preserves DNA integrity for long-term storage, while modified TENT suits short-term storage due to increased degradation. PowerPlex^®^ Fusion 6C and ForenSeq™ DNA Signature Prep kits present potential for forensic analysis using free DNA. Amplifying the mtDNA control region from free DNA highlights its potential for mtDNA heteroplasmy analysis. Additionally, free DNA is a valuable tool for quality control using ACTB and enables microbiome analysis via the 16S rRNA region for individual identification. Future research should validate and expand our findings to understand interactions affecting free DNA quantity comprehensively.

### Supplementary Information

Supplementary Information.

Supplementary Tables.

## Data Availability

All data supporting the findings of this study are available within the article and its supplementary materials.
