# Supplementary material for: Assessing the feasibility of free DNA for disaster victim identification and forensic applications
Source: Sci Rep. 2024 Mar 5;14:5411. doi: 10.1038/s41598-024-53040-0 (PMC10914783; doi:10.1038/s41598-024-53040-0)
Supplement: Supplementary file 1 — Supplementary Information. [file 41598_2024_53040_MOESM1_ESM.docx]

# Supplemental Material

**Assessing the feasibility of free DNA for disaster victim identification and forensic applications**

The supplemental material consists of Supplementary Figures S1-S5 and legends for Supplementary Tables S1-S9. The tables are provided in a separate Excel file.

**Supplementary Figures: pages 2-6**

**Supplementary Tables Legends: pages 7**

**Supplementary Tables: see separated Excel file**


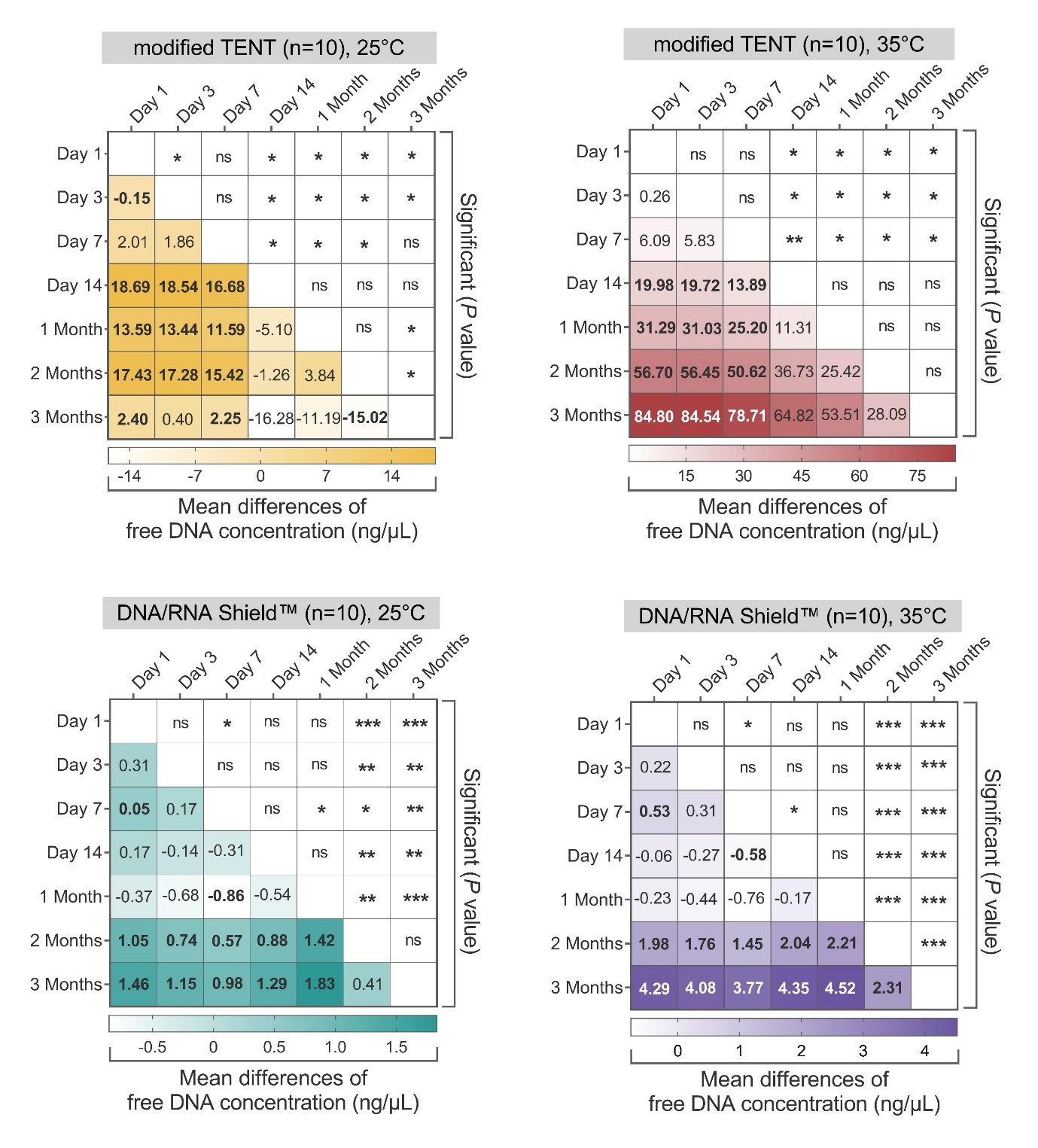


**Supplementary Figure S1**: Least Significant Difference (LSD) pairwise comparison test results of free DNA concentration in fresh tissues (n=10) under different conditions. The significance levels were set at p < 0.05(*), p < 0.01(**), and p < 0.001(***), ns = not significant, indicating significant differences between free DNA concentration at different time points.


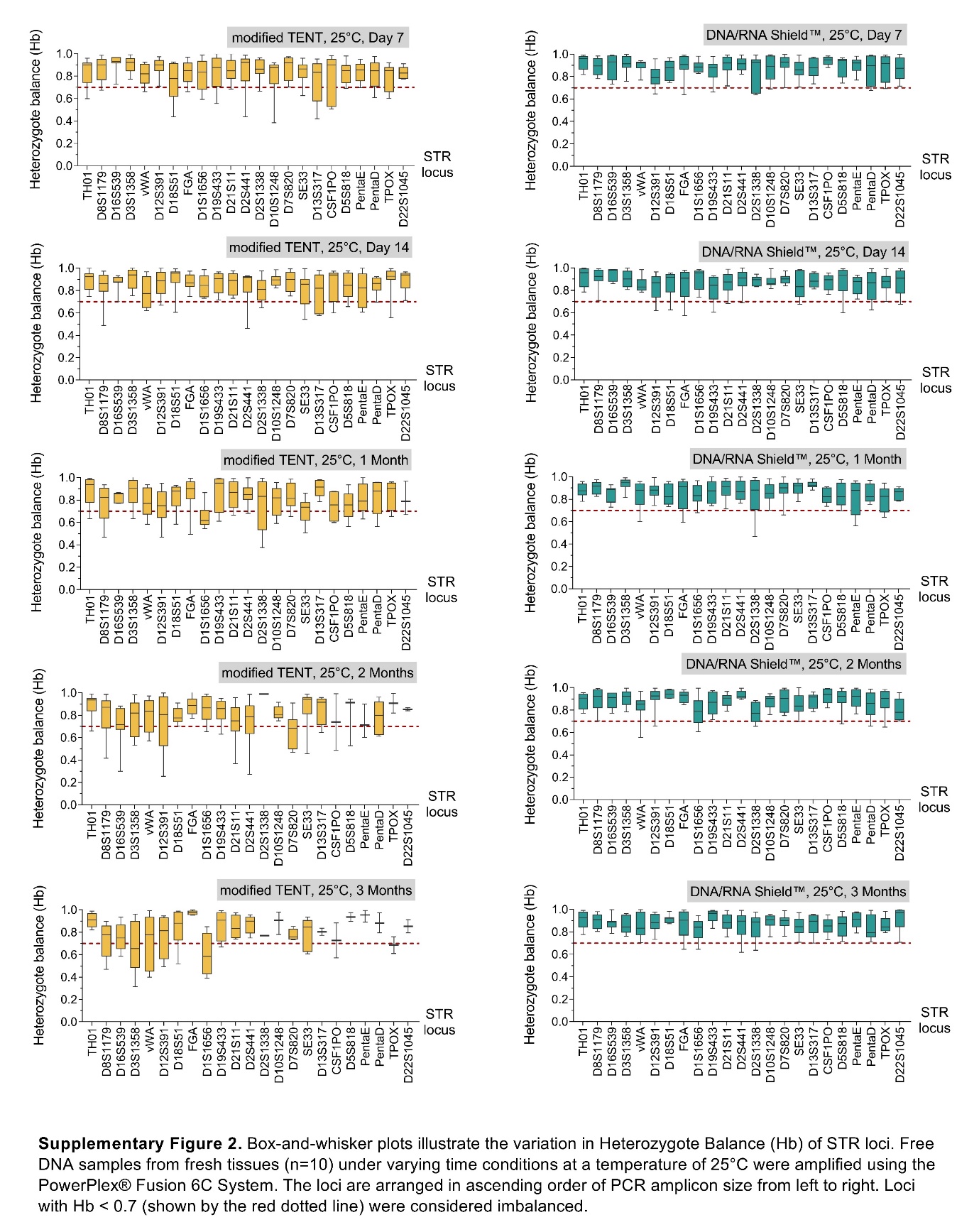


**Supplementary Figure S2**: Box-and-whisker plots illustrate the variation in Heterozygote Balance (Hb) of STR loci. Free DNA samples from fresh tissues (n=10) under varying time conditions at a temperature of 25°C were amplified using the PowerPlex® Fusion 6C System. The loci are arranged in ascending order of PCR amplicon size from left to right. Loci with Hb < 0.7 (shown by the red dotted line) were considered imbalanced.


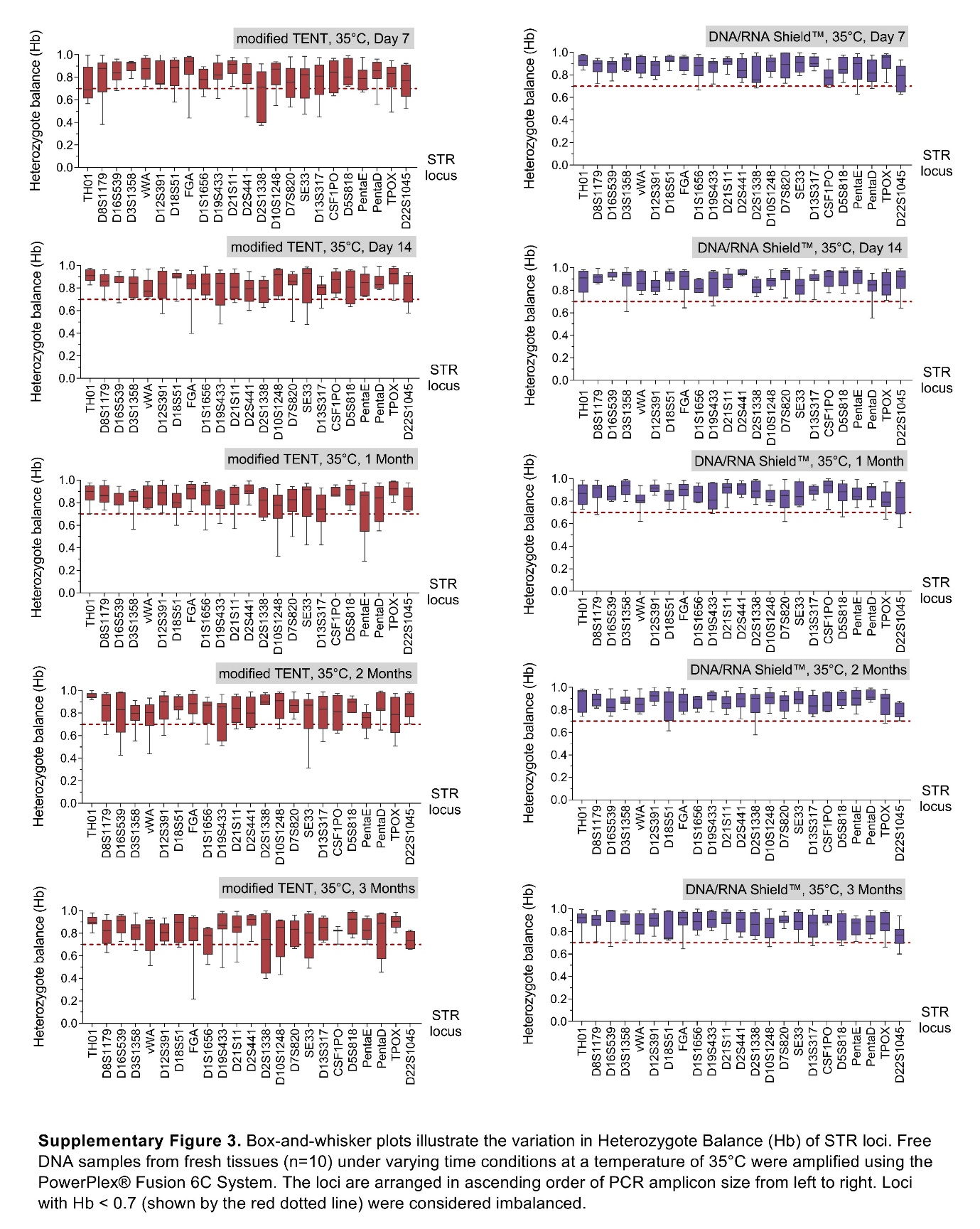


**Supplementary Figure S3**: Box-and-whisker plots illustrate the variation in Heterozygote Balance (Hb) of STR loci. Free DNA samples from fresh tissues (n=10) under varying time conditions at a temperature of 35°C were amplified using the PowerPlex® Fusion 6C System. The loci are arranged in ascending order of PCR amplicon size from left to right. Loci with Hb < 0.7 (shown by the red dotted line) were considered imbalanced.


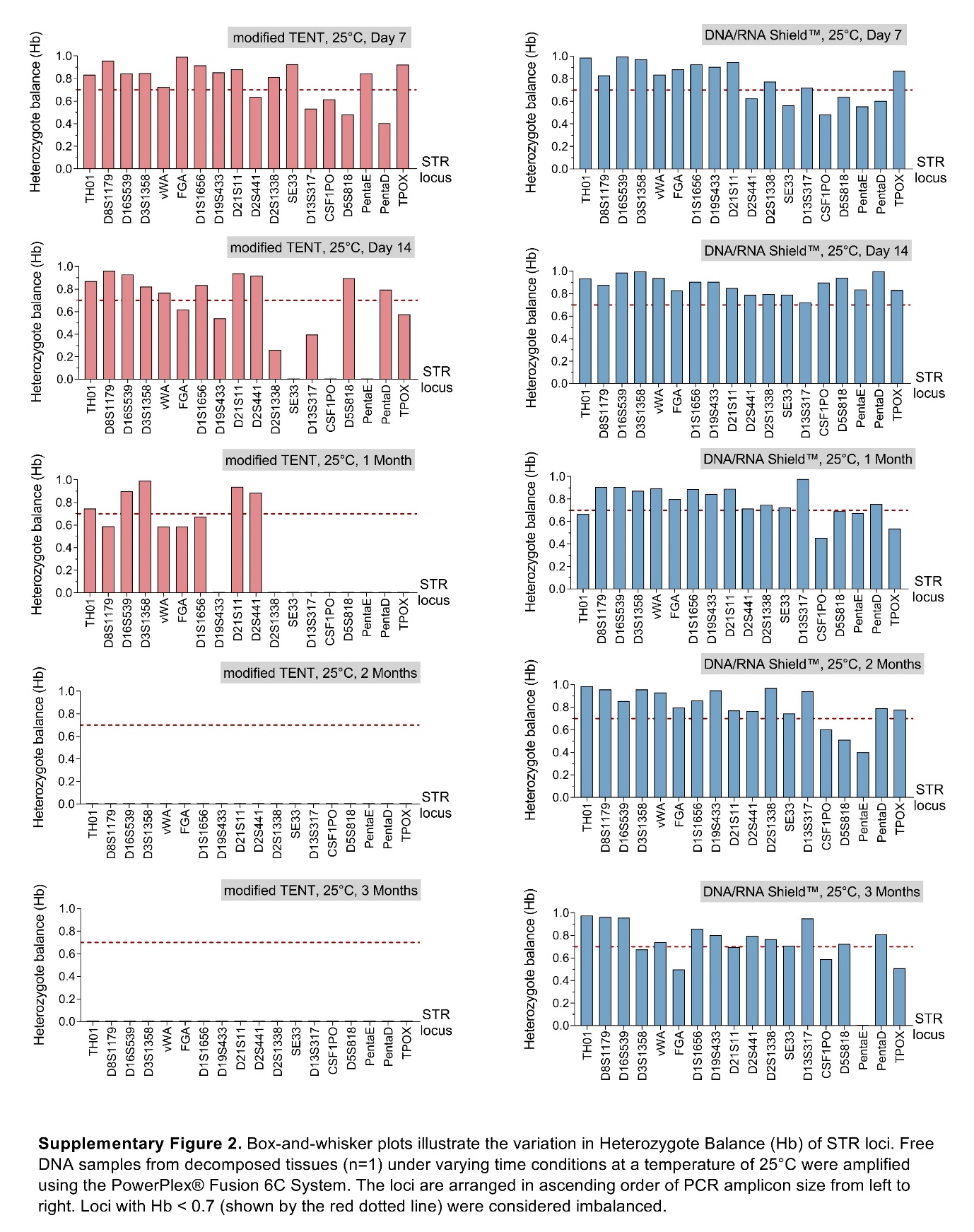


**Supplementary Figure S4**: Bar graph illustrate the variation in Heterozygote Balance (Hb) of STR loci. Free DNA samples from decomposed tissue (n=1) under varying time conditions at a temperature of 25°C were amplified using the PowerPlex® Fusion 6C System. The loci are arranged in ascending order of PCR amplicon size from left to right. Loci with Hb < 0.7 (shown by the red dotted line) were considered imbalanced.


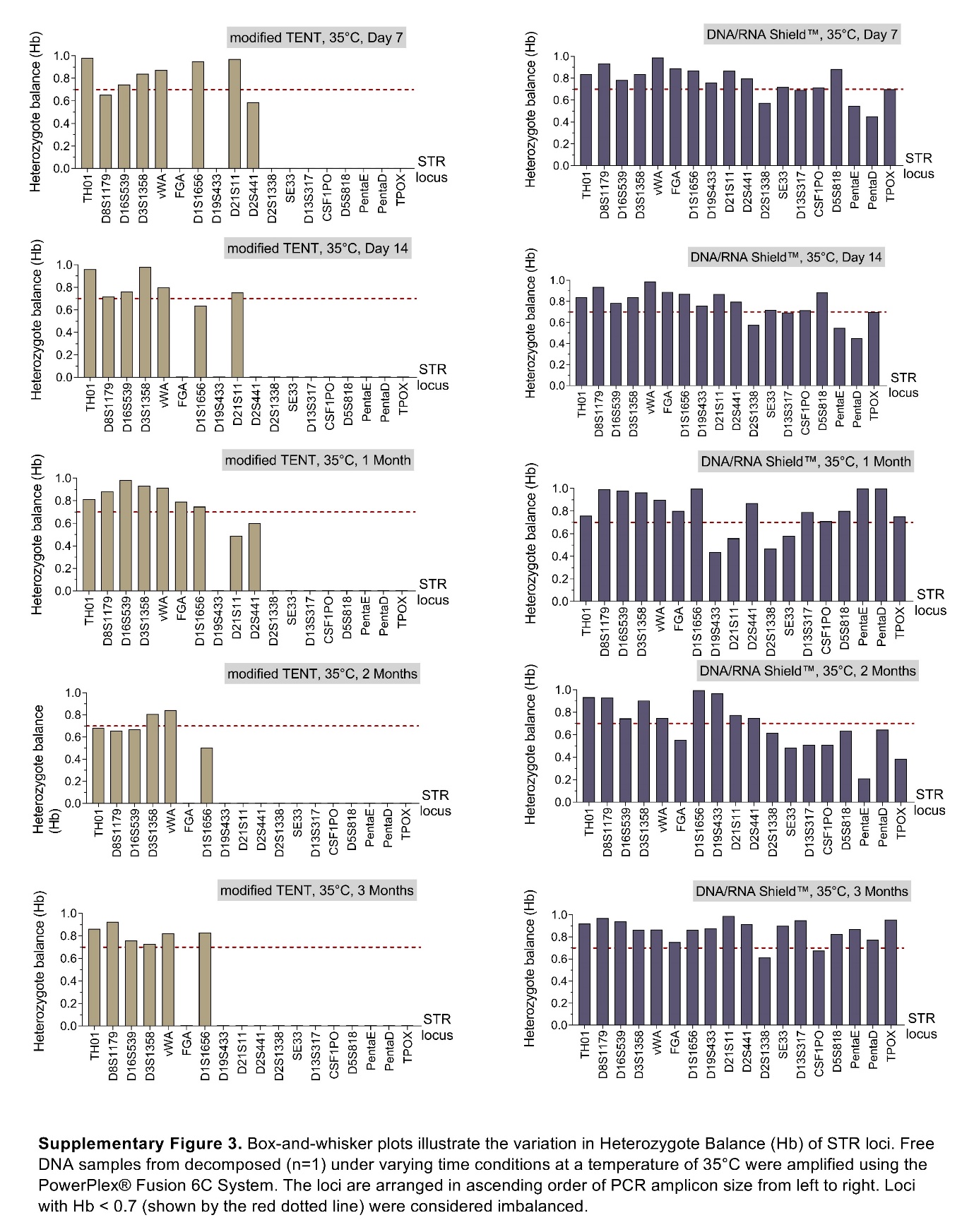


**Supplementary Figure S5**: Bar graph illustrate the variation in Heterozygote Balance (Hb) of STR loci. Free DNA samples from decomposed tissue (n=1) under varying time conditions at a temperature of 35°C were amplified using the PowerPlex® Fusion 6C System. The loci are arranged in ascending order of PCR amplicon size from left to right. Loci with Hb < 0.7 (shown by the red dotted line) were considered imbalanced.

**Supplementary Tables:**

Supplementary Table S1. Primers used for PCR amplification of human ACTB, 16S rRNA and ITS.

**Supplementary Table S2.** The statistical analysis of free DNA concentration from fresh tissues (n=10) under different conditions.

Pairwise comparisons were made using the Least Significant Difference method with a 95% confidence interval for differences. The significance levels were set at p < 0.05(*), p < 0.01(**), and p < 0.001(***), indicating significant differences between Group (I) and Group (J).

**Supplementary Table S3.** Comparison of alleles reports for the autosomal STR loci typed by PowerPlex® Fusion 6C and Verogen ForenSeq™ assay.

**Supplementary Table S4.** Comparison of the concordance and discordance (sample #9) for the autosomal STR loci typed by PowerPlex® Fusion 6C and Verogen ForenSeq™ assay.

**Supplementary Table S5.** Comparison of the concordance and discordance (sample #10) for the autosomal STR loci typed by PowerPlex® Fusion 6C and Verogen ForenSeq™ assay.

**Supplementary Table S6.** Comparison of the concordance and discordance (decomposed tissue sample) for the autosomal STR loci typed by PowerPlex® Fusion 6C and Verogen ForenSeq™ assay.

**Supplementary Table S7.** The comparison of the HV1 and HV2 results with the revised Cambridge Reference Sequence (rCRS).

**Supplementary Table S8.** C-stretch sequences of mtDNA HV1 and HV2 regions in each sample under various conditions.

**Supplementary Table S9.** A summary of PCR amplification results for human ACTB, 16S rRNA, and ITS in each free DNA conditions.
